# High-Resolution Free-Breathing Quantitative First-Pass Perfusion Cardiac MR Using Dual-Echo Dixon With Spatio-Temporal Acceleration

**DOI:** 10.3389/fcvm.2022.884221

**Published:** 2022-04-29

**Authors:** Joao Tourais, Cian M. Scannell, Torben Schneider, Ebraham Alskaf, Richard Crawley, Filippo Bosio, Javier Sanchez-Gonzalez, Mariya Doneva, Christophe Schülke, Jakob Meineke, Jochen Keupp, Jouke Smink, Marcel Breeuwer, Amedeo Chiribiri, Markus Henningsson, Teresa Correia

**Affiliations:** ^1^Department of Biomedical Engineering, Eindhoven University of Technology, Eindhoven, Netherlands; ^2^Department of MR R&D – Clinical Science, Philips Healthcare, Best, Netherlands; ^3^Department of Imaging Physics, Magnetic Resonance Systems Lab, Delft University of Technology, Delft, Netherlands; ^4^School of Biomedical Engineering and Imaging Sciences, King's College London, London, United Kingdom; ^5^Philips Healthcare, Guildford, United Kingdom; ^6^Philips Healthcare Iberia, Madrid, Spain; ^7^Philips Research, Hamburg, Germany; ^8^Division of Cardiovascular Medicine, Department of Medical and Health Sciences, Linkoping University, Linkoping, Sweden; ^9^Center for Medical Image Science and Visualization (CMIV), Linkoping University, Linkoping, Sweden; ^10^Centre for Marine Sciences (CCMAR), Faro, Portugal

**Keywords:** myocardial perfusion, high-resolution, free-breathing, quantitative myocardial blood flow, Dixon, motion correction

## Abstract

**Introduction:**

To develop and test the feasibility of free-breathing (FB), high-resolution quantitative first-pass perfusion cardiac MR (FPP-CMR) using dual-echo Dixon (FOSTERS; Fat-water separation for mOtion-corrected Spatio-TEmporally accelerated myocardial peRfuSion).

**Materials and Methods:**

FOSTERS was performed in FB using a dual-saturation single-bolus acquisition with dual-echo Dixon and a dynamically variable Cartesian k-t undersampling (8-fold) approach, with low-rank and sparsity constrained reconstruction, to achieve high-resolution FPP-CMR images. FOSTERS also included automatic in-plane motion estimation and T${}_{2}^{*}$ correction to obtain quantitative myocardial blood flow (MBF) maps. High-resolution (1.6 x 1.6 mm^2^) FB FOSTERS was evaluated in eleven patients, during rest, against standard-resolution (2.6 x 2.6 mm^2^) 2-fold SENSE-accelerated breath-hold (BH) FPP-CMR. In addition, MBF was computed for FOSTERS and spatial wavelet-based compressed sensing (CS) reconstruction. Two cardiologists scored the image quality (IQ) of FOSTERS, CS, and standard BH FPP-CMR images using a 4-point scale (1–4, non-diagnostic – fully diagnostic).

**Results:**

FOSTERS produced high-quality images without dark-rim and with reduced motion-related artifacts, using an 8x accelerated FB acquisition. FOSTERS and standard BH FPP-CMR exhibited excellent IQ with an average score of 3.5 ± 0.6 and 3.4 ± 0.6 (no statistical difference, *p* > 0.05), respectively. CS images exhibited severe artifacts and high levels of noise, resulting in an average IQ score of 2.9 ± 0.5. MBF values obtained with FOSTERS presented a lower variance than those obtained with CS.

**Discussion:**

FOSTERS enabled high-resolution FB FPP-CMR with MBF quantification. Combining motion correction with a low-rank and sparsity-constrained reconstruction results in excellent image quality.

## Introduction

First-pass perfusion cardiac MR (FPP-CMR) enables non-invasive detection of ischemic heart disease (1–3). Typically, assessment is based on visual comparison of relative contrast enhancement in different myocardial segments, which requires highly trained readers (4). Quantitative FPP-CMR (QFPP-CMR) provides an objective assessment by estimating pixel-wise myocardial blood flow (MBF) (5) and has high diagnostic and prognostic value (6–10). However, there are several technical challenges that can negatively impact the image quality and the diagnostic yield. Since MBF quantification is based on modeling the signal intensity during the first pass of a contrast agent bolus, sources of motion must be minimized to ensure that the same anatomy is depicted for a given pixel across time. In particular, the duration of the first pass, approximately 30–50 s, does not fit into a breath-hold and therefore, respiratory motion poses a significant challenge (11). Free-breathing QFPP-CMR can be performed with retrospective respiratory motion correction using image registration, yet the localized strong image contrast changes can hamper the performance of conventional signal intensity-based registration algorithms (12, 13). FPP-CMR images are also commonly affected by the dark-rim artifact which mimics perfusion defects and is exacerbated by a low spatial resolution (14, 15). The signal from subcutaneous, epicardial, or intramyocardial fat may also adversely impact quantification and image quality. While fat-selective saturation prepulses can be employed, in practice they are limited to centric phase encoding sampling which can cause blurring and reduced contrast. Finally, MBF quantification may be biased by signal nonlinearities at very high contrast agent concentrations due to T_1_ saturation and T${}_{2}^{*}$-related signal loss (5, 16–18).

Recently, a dual-bolus multi-echo Dixon QFPP-CMR framework has been proposed to address in-plane respiratory motion, T${}_{2}^{*}$-related signal loss, and fat suppression (19). This method provides fat-only images, which were used to estimate respiratory motion, while motion-corrected water-only images were used for visual assessment and MBF quantification. In this work, a framework titled “Fat-water separatiOn for motion-corrected Spatio-TEmporally accelerated myocardial peRfuSion” (FOSTERS) is proposed. FOSTERS extends the previous work by combining a dynamic variable undersampled dual-echo Dixon acquisition with a motion-corrected reconstruction with low-rank and sparsity constraints to achieve high-resolution FPP-CMR images. Additionally, the high-resolution acquisition is interleaved with a low-resolution image with a low T_1_ sensitivity for estimating the arterial input function (AIF) (20). As before, echo images were used for correcting the AIF for T${}_{2}^{*}$-related signal losses to further improve MBF quantification. The performance of FOSTERS is compared to a standard compressed sensing reconstruction as well as the corresponding clinical standard-resolution breath-hold FPP-CMR. This comparison assessed the variability of MBF, the image sharpness, and the image quality scores of expert readers.

## Materials and Methods

### FOSTERS Framework

#### Pulse Sequence

**Figure 1:**
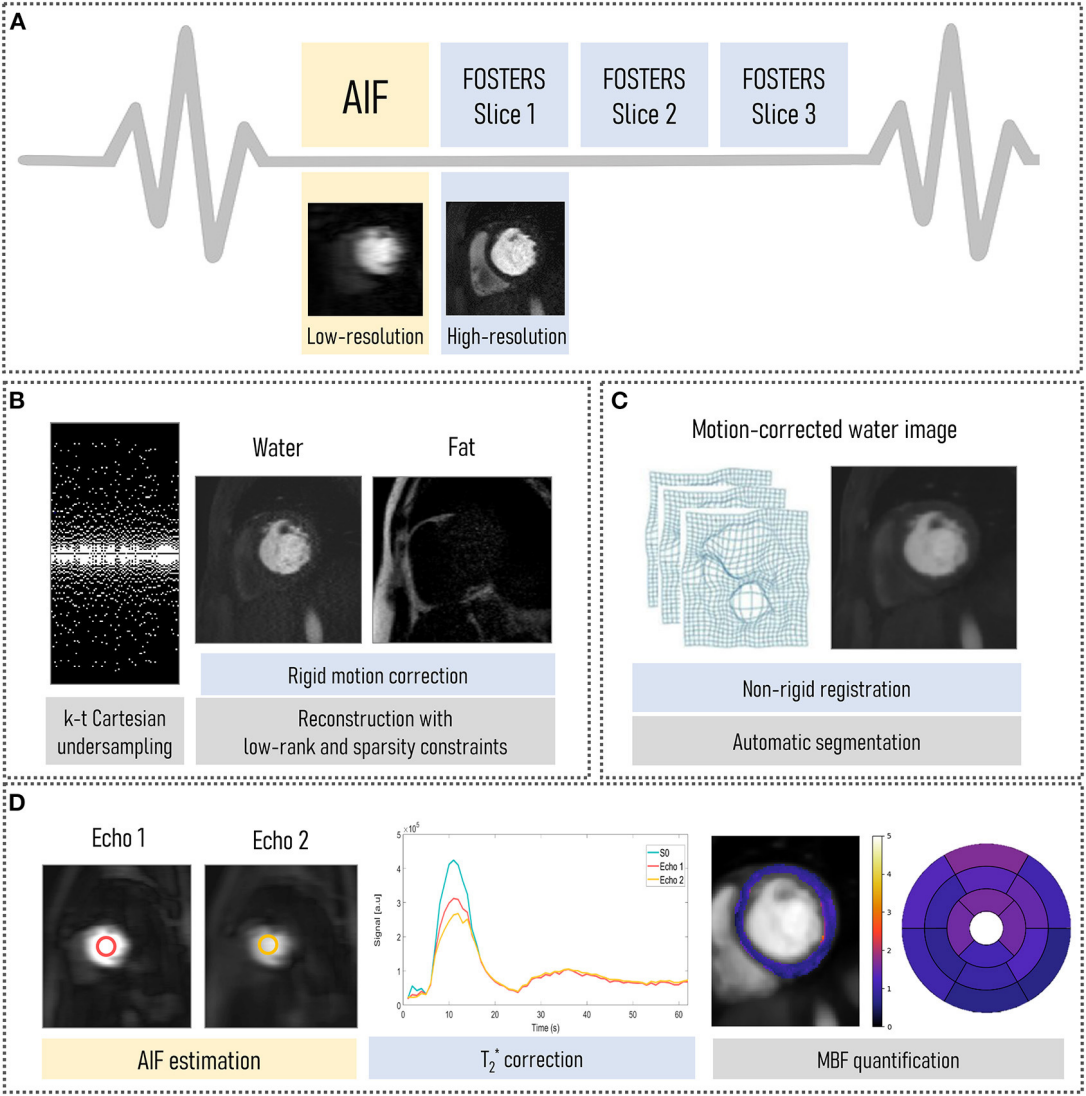
FOSTERS framework: **(A)** A dual-saturation dual-echo Dixon FPP-CMR sequence was used to acquire low-resolution arterial input function (AIF) and high-resolution myocardial images. **(B)** Water- and fat-only images were obtained from k-t undersampled data using a fast low-rank and sparsity constrained reconstruction method with 10 iterations. Fat-only images were used to estimate in-plane respiratory motion. Then, rigidly motion-corrected images were generated using the same fast low-rank and sparsity regularized reconstruction method with 50 iterations. **(C)** The rigidly motion-corrected water-only images were fine-tuned using non-rigid registration and were automatically segmented to an AHA 16-segment model. **(D)** AIF echoes were used to correct for T2* decay and quantitative myocardial blood flow (MBF) maps were automatically obtained.

The FOSTERS pipeline (shown in Figure 1) comprises an electrocardiogram-triggered multi-slice dual-saturation (21) single-bolus acquisition with dual-echo gradient-echo imaging to allow for water-fat separation and T${}_{2}^{*}$ correction. In each cardiac cycle, to measure the AIF, the dual-echo acquisition is preceded by a low-resolution image with a short saturation time (20). A variable density Poisson distribution undersampled Cartesian acquisition was employed (22), where the center of k-space is more densely sampled than the periphery, to achieve an incoherent artifact distribution. In addition, the k_y_ pattern was pseudo-randomly varied individually for each time point (k-t acceleration).

#### Image Reconstruction and Motion Correction

The FOSTERS image reconstruction and motion correction were implemented in the Recon 2.0 environment (Philips, Best, The Netherlands) to allow for inline scanner integration. First, a Dixon reconstruction with compressed sensing (CS) using low-rank (time-domain) and sparsity (spatial domain) constraints (23) was performed with 10 iterations (empirically determined), which generates water- and fat-only images from k-t undersampled data with sufficient quality for in-plane respiratory motion estimation using image registration.

Thresholding, followed by dilation, was then employed to create a binary mask from the water images. From this binary mask, a bounding box was automatically placed around the epicardial fat and was used to estimate rigid respiratory motion using the Fast Elastic Image Registration (FEIR) toolbox (24) with normalized gradient fields as an image similarity measure. Fat images were used as a stable reference for the anatomy because they do not show contrast uptake-related image intensity changes, as proposed by Scannell et al. (19). A reference time frame, with superior-inferior (SI) motion displacement closest to the mean SI position, was selected. This translational motion information was used to correct rigidly the dual-echo data by applying a linear phase shift in k-space. Rigid motion only was estimated in this step due to the sparse signal of the fat images being unsuitable for non-rigid motion estimation. A non-rigid refinement step is performed at a later stage.

Finally, motion-corrected water-only images were generated using a CS reconstruction with low-rank and sparsity constraints after 50 iterations. Images were reconstructed using a fast sparsity and nuclear norm regularization method (23), which solved the following minimization problem:


$$\hat{x}=\arg\min_{x}\{\frac{1}{2}{\left\|Ex-k\right\|}_{2}^{2}+\alpha {\left\|x\right\|}_{*}+\beta {\left\|\Psi x\right\|}_{1}\},$$

where *x* are the dynamic images, *k* is the dynamic time-series data (after translational motion correction), *E* is the SENSE encoding operator, Ψ is the spatial anisotropic total variation operator, α and β are regularization parameters, || ∙ ||_*_ is the nuclear norm (sum of singular values) and || ∙ ||_1_ is the L1-norm. The regularization parameters were selected empirically and set at α = 1 and β = 0.005 for all subjects.

#### Post-processing: T${}_{2}^{*}$ Correction and Quantification of Myocardial Blood Flow

The rigidly motion-corrected dynamic water-only images were fine-tuned using non-rigid registration to a corresponding motionless synthetic image series, generated with principal component analysis (12). FPP-CMR images were automatically segmented to an AHA 16-segment model using a deep learning-based method, as previously published (25). This model comprises four neural networks applied sequentially: a convolutional neural network (CNN) to detect the time-frame with peak left ventricle (LV) enhancement, a CNN to select a bounding box that encompasses the LV cavity and myocardium, a U-Net to segment the myocardium, and a U-Net to detect the right ventricle insertion points that define the 16 AHA-segments. In addition, the dual-echo images were used for estimating the AIF and T${}_{2}^{*}$-related signal loss by fitting the mean signal magnitude to an exponential decay model (19). Quantitative MBF values were estimated on a pixel-wise level by fitting the observed AIF and myocardial tissue curves to a two-compartment exchange model, using Bayesian inference (26). MBF quantification was performed using only the dynamic contrast-enhanced data corresponding to the first pass of the contrast bolus (approximately 20 sec of data).

### *In vivo* Experiments

All acquisitions reported in this study were performed on a 3.0T Achieva scanner (Philips, Best, The Netherlands) using a 32-channel cardiac coil. The study was approved by the National Research Ethics Service (15/NS/0030) and written informed consent was obtained from each participant according to institutional guidelines. All the patients enrolled in this study underwent a CMR examination for clinical nonstress function and viability assessment with known or suspected heart disease. Patients were required to be ≥18 years of age and have no contraindications to gadolinium contrast, inclusive of an estimated glomerular filtration rate ≤ 60 ml/min/1.73 m^2^.

Eleven patients (baseline characteristics in Supplementary Table 1) with suspected cardiovascular disease were scanned during rest with 8-fold k-t accelerated FOSTERS during the first pass of a contrast bolus injection (0.075 mmol/kg of Gadobutrol at 4 ml/s followed by 25 ml saline flush). Three short-axis slices (basal, mid, and apical) were acquired with the following parameters: FOV = 320 × 300 mm^2^, matrix size = 200 × 186, acquired/reconstructed in-plane resolution = 1.6 × 1.6 / 1.43 x 1.43 mm^2^, slice thickness = 10 mm, TR/TE1/TE2 = 2.8/1.1/1.9 ms, acceleration factor (R) = 8, saturation time (short TS / long TS) = 23.5/100 ms, flip angle = 15°, acquisition window = 65.4 ms, temporal resolution = 145 ms, bandwidth = 2,083.3 Hz, 54–87 dynamic frames, and scan time = 60 s. Apart from the previously mentioned saturation time and acquired in-plane resolution, all imaging parameters were kept constant between the AIF and the dual-echo images. The dual-echo FPP-CMR datasets were also reconstructed with the vendor's commercially available inline CS wavelet-based reconstruction (only spatial sparsity constraints with the default parameters) (27) and non-rigid motion correction. A BH standard-resolution 2D FPP-CMR acquisition (referred here as standard BH) (20) was acquired for the same eleven subjects with identical imaging parameters to FOSTERS except for in-plane resolution = 2.6 × 2.6 mm^2^, TR/TE = 2.2/1 ms, temporal resolution = 160 ms, SENSE = 2 and partial Fourier = 0.75. The standard BH and FOSTERS scans were performed with individual contrast injections and were separated by 5–7 min to allow for contrast washout.

To assess the motion correction performance of FOSTERS in different circumstances, one patient (baseline characteristics in Supplementary Table 1) was scanned separately with FOSTERS both in free-breathing (FB) and breath-hold (BH) during the same CMR examination. The scan parameters were kept identical for both acquisitions, and the BH-FOSTERS was acquired 11 min after the FB-FOSTERS.

### Image Evaluation and Statistical Analysis

FOSTERS, CS, and standard BH were processed, including the non-rigid motion compensation, following the procedure described in section 2.1.3. The MBF values estimated with FOSTERS were compared to those obtained with CS. The FOSTERS MBF values were not compared directly with the standard BH MBF values as the standard BH acquisition was the second contrast injection and the MBF values are biased by the residual contrast from the first injection. The presence of dark-rim artifacts and the image quality (IQ) of the three slices acquired with FOSTERS, CS, and standard BH were assessed jointly by two experienced cardiologists in a randomized setup, blinded to the patient information and imaging. IQ was graded on a scale of 1 to 4, in consensus: where (1) was non-diagnostic IQ; (2) was diagnostic IQ with major artifacts; (3) diagnostic IQ with minor artifacts; and (4) was fully diagnostic IQ with no artifacts. For each dataset (FOSTERS, CS, and standard BH) the total combined IQ score was calculated as the average score of the three slices. Quantitative image sharpness was calculated for FOSTERS, CS, and standard BH. For each patient, the three acquired slices were selected for sharpness analysis. In each image, a profile was manually selected between the left ventricle blood pool and the endocardium, as shown in Supplementary Figure 1. The sharpness was defined as the distance in pixels between 20% and 80% of the pixel intensity range of the profile, and a lower pixel distance indicates a sharper border (28). For FOSTERS, CS, and standard BH, the total image sharpness was calculated as the average of the three slices. For all statistical comparisons a *p*-value cut-off level of 0.05 was chosen to indicate significance and was performed using the Wilcoxon signed-rank test (IQ) and the Mann-Whitney U-test (sharpness).

## Results

**Figure 2:**
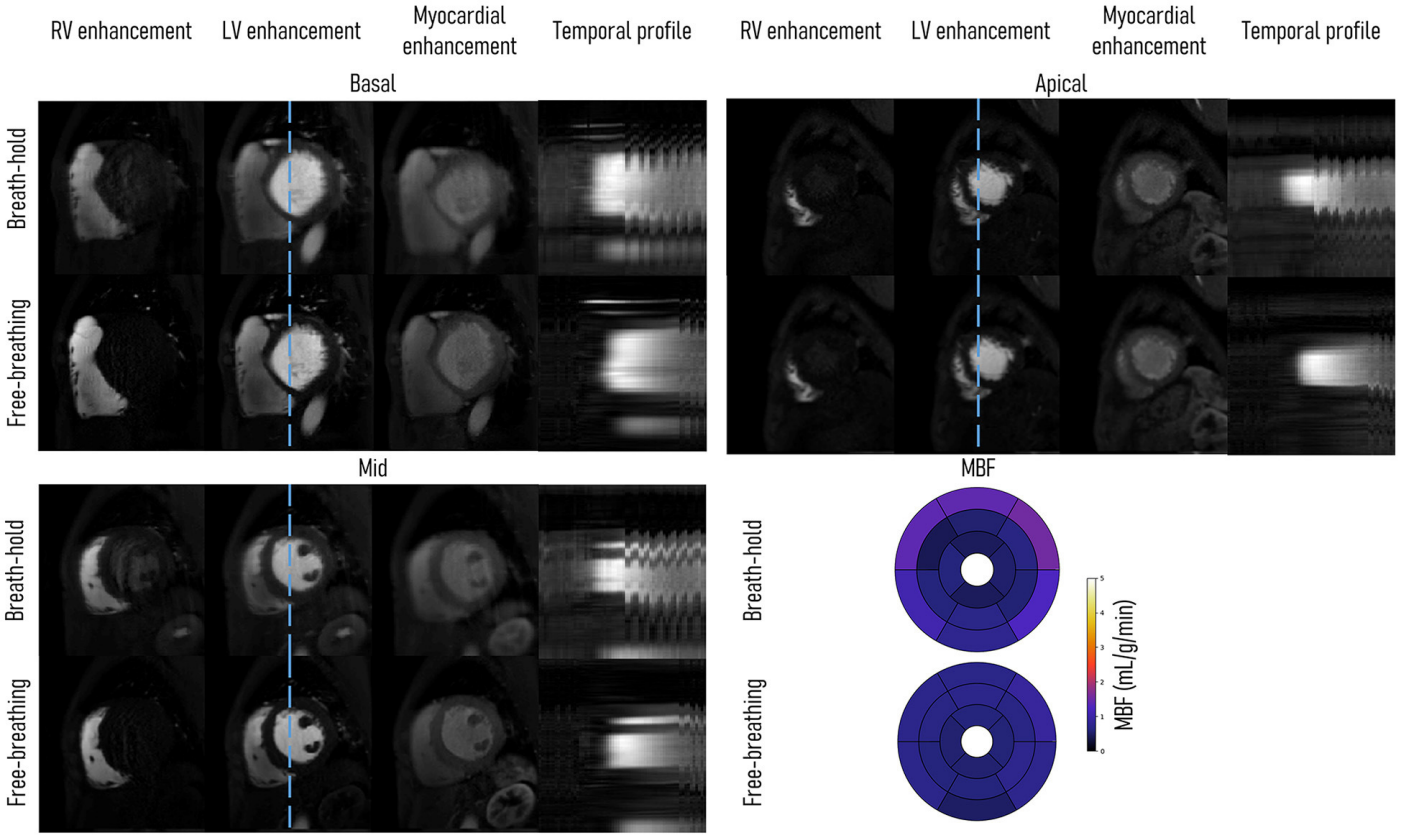
Single patient (diagnosed with dilated cardiomyopathy) comparison between breath-hold (BH) and free-breathing (FB) FOSTERS. Right ventricle (RV), left ventricle (LV), myocardial enhancement timeframes, and a temporal profile (blue dashed line) are displayed for the acquired three short-axis slices (basal, mid, and apical). FB-FOSTERS images exhibit excellent quality with no visible motion artifacts, despite some visible motion in the final part of the acquisition, as shown in the temporal profile. In some timeframes, BH-FOSTERS displays residual ghosting artifacts, due to unsuccessful motion correction. This can be explained by the more regular respiratory motion during FB which is easier to correct than the large amplitude motion that may occur due to incomplete breath-holding. The 16-segment bullseye plot shows that the myocardial blood flow (MBF) values were more uniform for FB- than for BH-FOSTERS (average ± SD for the 16 segments of 0.7 ± 0.1 and 0.8 ± 0.4 mL/min/g, respectively). The reconstruction parameters were kept identical for both approaches.

Figure 2 shows the comparison of FB and BH FOSTERS on a single patient. Three-time frames are displayed for each slice together with 16-segment bullseye plots. Good image quality was achieved with both approaches, with FB-FOSTERS exhibiting overall sharper image features, while BH-FOSTERS presented some residual ghosting artifacts in some timeframes. In addition, FB-FOSTERS (0.7 ± 0.1 mL/min/g) yielded MBF values with 4-fold lower variation when compared to BH-FOSTERS (0.8 ± 0.4 mL/min/g).

**Figure 3:**
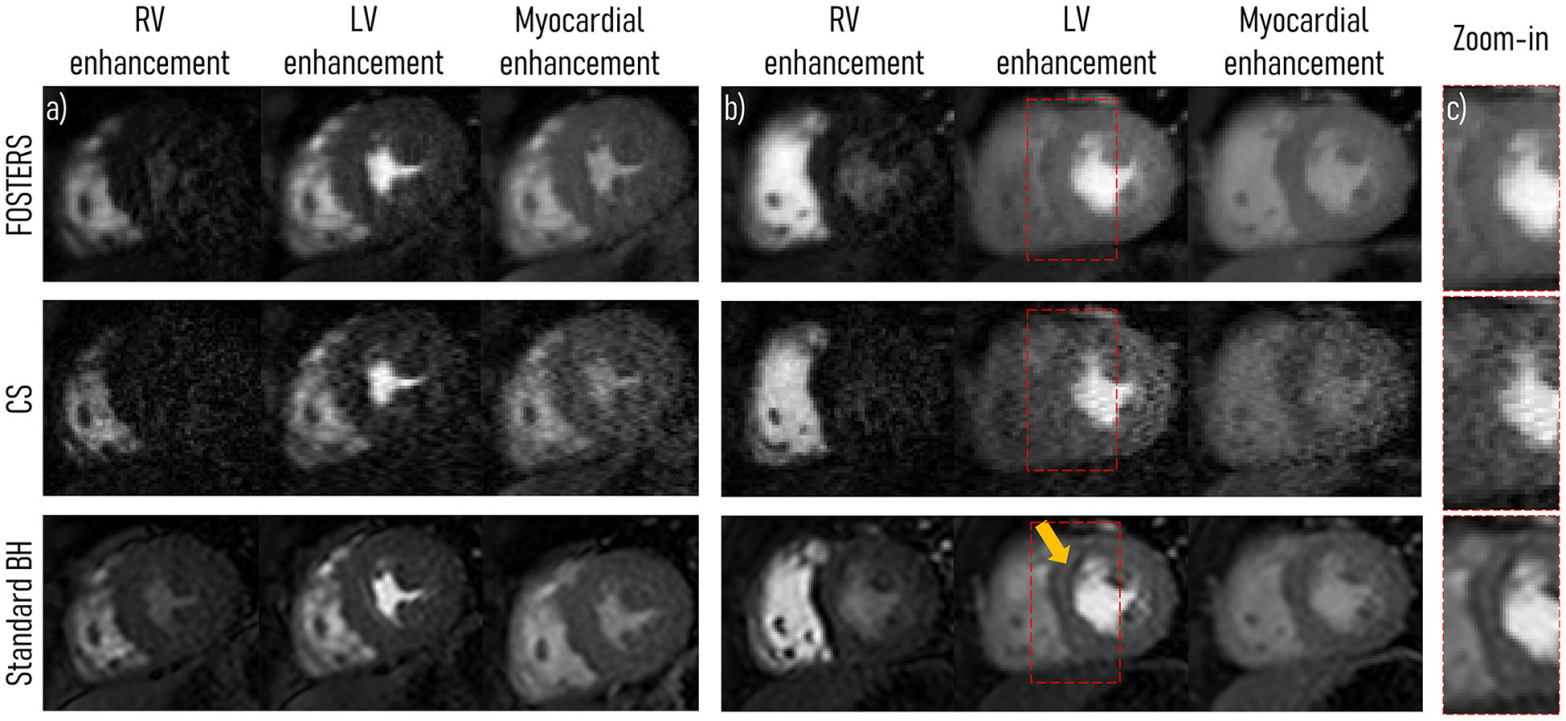
A single short-axis view at mid-ventricular level is displayed during right ventricle (RV), left ventricle (LV), and myocardial enhancement for two representative patients (middle slice for **(A)** patient 5 and **(B)** 7). High-resolution free-breathing water-only FPP-CMR FOSTERS and spatial wavelet-based compressed sensing (CS) reconstruction in addition to standard-resolution FPP-CMR (Standard BH) are displayed. Overall, CS FPP-CMR exhibits a higher level of noise and artifacts compared to FOSTERS and standard BH. **(C)** In the zoom-in region (red rectangle), a dark-rim artifact can be seen in the standard BH images (arrow), which were not visible in the FOSTERS and CS images. Supplementary Videos 1, 2 contains an animation of these datasets for all approaches.

All 11 patient scans were completed, reconstructed, and the in-plane motion was estimated successfully for all slices. Figure 3 displays a comparison between the water-only images (middle slice) obtained with FB-FOSTERS, spatial wavelet-based CS, and standard BH for two representative patients (patients 5 and 7). Supplementary Videos 1, 2 contain animations of these datasets for all approaches.Three different FOSTERS timeframes are displayed, demonstrating high image quality with no visible motion artifacts and clear myocardial depiction. Conversely, CS exhibited degraded image quality with high levels of noise. These differences between FOSTERS and CS are also visible in the fat-only images and the temporal profile as shown in Supplementary Figure 2 and Supplementary Video 3. Standard BH FPP-CMR achieved excellent image sharpness (Figure 3), but dark-rim artifacts were still present in 7 out of the 33 cases (3 acquired slices for 11 patients). These were not visible in the FOSTERS and CS images.

**Figure 4:**
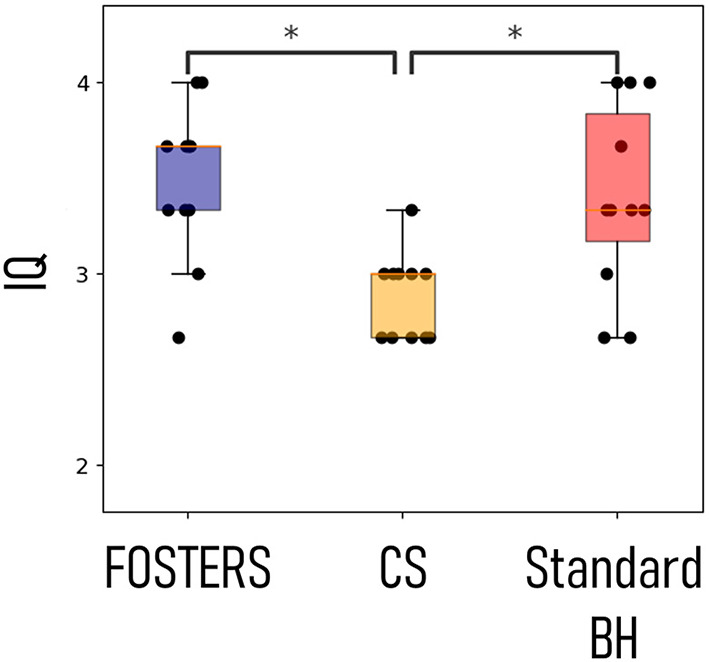
Image quality (IQ) scores for the eleven patients. The three acquired slices were independently scored in terms of image quality for the high-resolution FPP-CMR (FOSTERS and CS) and the standard resolution (Standard BH), and the values were averaged for each patient. Statistically significant differences (*p* < 0.05) are indicated by *.

Figure 4 shows the image quality score for all the patients, as an average score for the three slices, using FB-FOSTERS, CS, and standard BH. FOSTERS scored the highest (3.5 ± 0.6), followed by standard BH (3.4 ± 0.6) and CS (2.9 ± 0.5). The differences between FOSTERS and CS and between standard BH and CS were statistically significant (*p* = 0.004 and 0.02, respectively). There was no significant difference between the FOSTERS and standard BH images (*p* = 0.72). The mean blood-myocardium sharpness ± standard deviation was 4.8 ± 1.8 for FOSTERS, 3.2 ± 2.1 for CS, and 4.9 ± 2.4 for standard BH. For the blood-myocardium sharpness measurements, no statistically significant differences were found between FOSTERS and CS (*p* = 0.08), between FOSTERS and standard BH (*p* = 0.68), and between CS and standard BH (*p* = 0.25).

**Figure 5:**
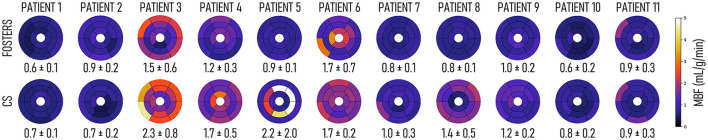
16-segment myocardial blood flow (MBF) plots for the high-resolution FB FOSTERS and compressed sensing (CS) reconstruction approaches for all eleven patients. Mean MBF ± SD for all segments is displayed below each plot. Overall, FOSTERS provides more homogenous MBF maps compared to CS, which can be explained by the lower residual respiratory motion artifacts present in the FOSTERS images.

**Figure 6:**
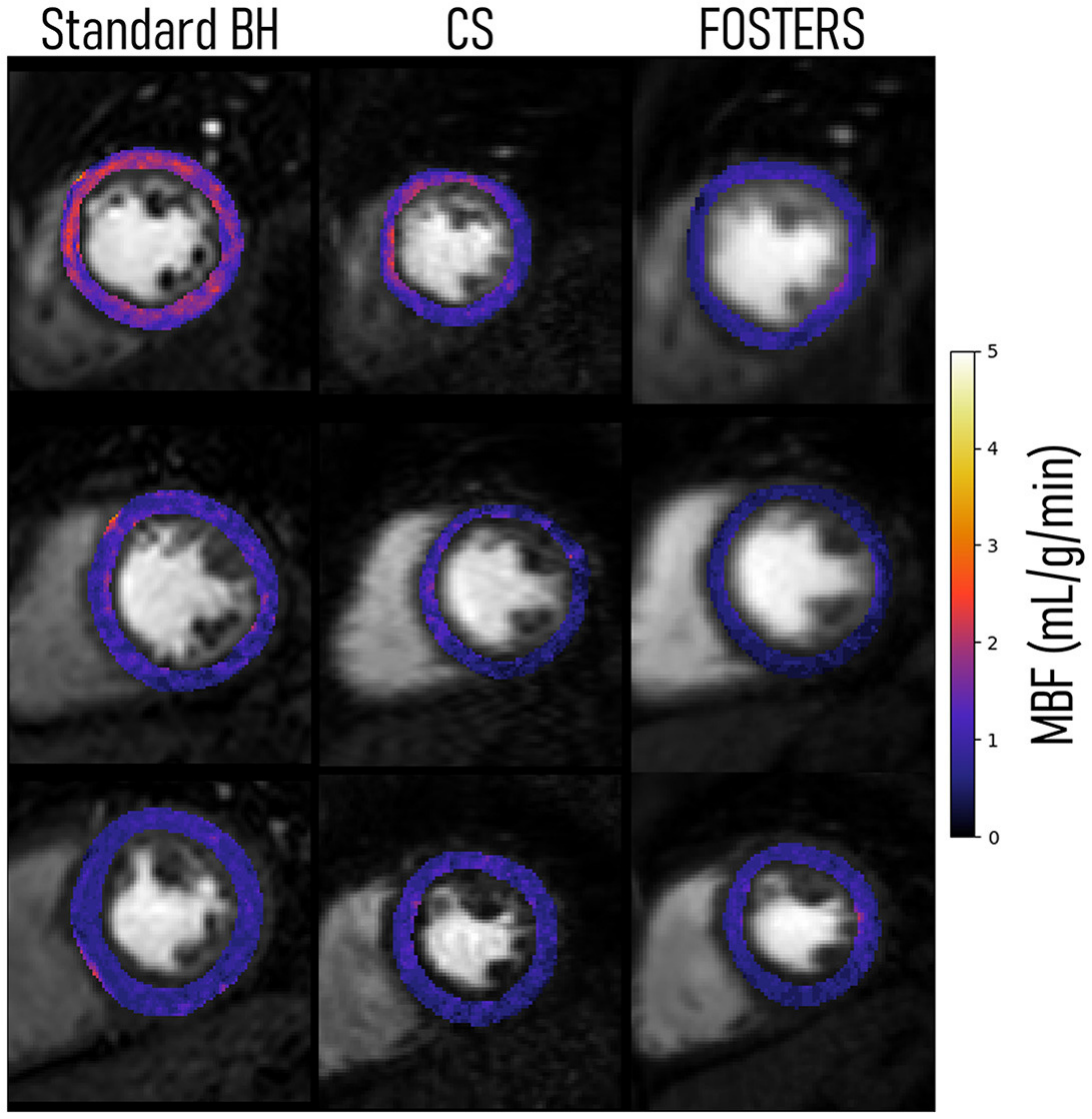
Pixel-wise myocardial blood flow (MBF) maps of a single slice (mid position) for three representative patients were obtained with Standard BH, CS, and FOSTERS. FOSTERS resulted in more homogenous MBF values, while CS and Standard BH exhibited a higher variance.

The 16-segment MBF plots for the FB-FOSTERS and CS approaches for all eleven patients are displayed in Figure 5. FOSTERS provides uniform MBF maps whereas CS results in MBF values with higher variation, which could be attributed to residual artifacts unresolved by the reconstruction algorithm and high levels of noise. These artifacts will affect the motion estimation performance, T${}_{2}^{*}$ correction of the AIF, and accuracy of the MBF estimation. The mean MBF (± SD) values were 1.0 (± 0.3) and 1.3 (± 0.6) mL/min/g for FOSTERS and CS, respectively. FOSTERS also resulted in a lower SD (0.4 mL/min/g) when compared to CS (0.6 mL/min/g). Significant differences (p = 0.01) in MBF were found between the two methods. Figure 6 shows representative slices of the pixel-wise MBF maps acquired with Standard BH, CS, and FOSTERS in three subjects. When compared to FOSTERS, the MBF maps obtained with Standard BH and CS exhibited a higher level of noise, resulting in a larger variation in the MBF values. MBF values obtained with the standard BH are higher due to the residual contrast from the FOSTERS acquisition, this is visible in the first row of Figure 6.

## Discussion

Here, the feasibility of high-resolution QFPP-CMR imaging during FB was demonstrated by using a dual-saturation dual-echo Dixon water-fat separation, a compressed sensing reconstruction with low-rank and sparsity constraints, and respiratory motion correction. The dynamically varying 8-fold Cartesian k-t undersampling allowed to obtain a short temporal resolution (< 150 ms) while maintaining the desired high in-plane resolution (1.6 × 1.6 mm^2^), making FOSTERS suitable for patients with heart rates up to 110 bpm. High-spatial resolution imaging is beneficial for minimizing dark-rim artifacts and detecting subtle sub-endocardial ischemia associated with coronary microvascular dysfunction (29) and is likely to improve the diagnostic yield of the modality.

As a proof-of-principle, to assess the performance of FOSTERS respiratory motion correction and MBF quantification, a comparison between FB- and BH-FOSTERS was performed in one patient (Figure 2). Fat-only images produced by the dual-echo Dixon acquisition allowed for accurate in-plane motion estimation during free-breathing. Moreover, FB-FOSTERS resulted in FPP-CMR images with excellent sharpness of the cardiac structures and more uniform MBF maps compared to BH-FOSTERS. This can be explained by an incomplete BH, which resulted in high MBF values observed in the anterior and lateral walls of the basal slices.

In the image quality evaluation, FB-FOSTERS was ranked the highest. In the representative cases displayed in Figure 3, FOSTERS presented an excellent depiction of the myocardium with minimal residual motion-related artifacts in the FPP-CMR images. Standard BH also exhibited excellent image quality, but the need for the BH significantly impacted the subject's comfort, which can lead to images with insufficient diagnostic quality. In addition, dark-rim artifacts were observed in standard BH images but were not visible in FOSTERS water-only FPP-CMR images because of the higher image resolution of FOSTERS compared to standard BH (1.6 × 1.6 and 2.6 × 2.6 mm^2^, respectively). The image quality using spatial wavelet-based CS was scored the lowest due to the high level of image artifacts and noise. On the other hand, image quality of k-t undersampled reconstruction is known to be sensitive to respiratory motion, which negatively affects the spatio-temporal correlations. The addition of a rigid motion correction step (translational motion) in combination with a non-rigid motion correction results in robust FB acquisitions, as previously demonstrated by Scannell et al. (19).

Overall, FOSTERS provided more homogenous MBF maps compared to CS for the eleven patients included in this work. This may be due to the higher respiratory motion artifacts present in the CS images, which result in higher values and variance in the measured MBF.

Compared to the multi-echo Dixon QFPP-CMR introduced by Scannell et al. (19), FB-FOSTERS is substantially accelerated using k-t undersampling and acquiring two, rather than three, echo images. Shortening the acquisition time window is necessary to allow the acquisition of three high-resolution slices in the short RR intervals associated with (stress) FPP-CMR imaging and allows higher in-plane spatial resolution. In addition, a dual-saturation strategy was employed, rather than a dual-bolus, allowing for the acquisition of AIF and myocardial tissue information in the same cardiac cycle and after injection of a single bolus.

Several other approaches have been proposed to accelerate FPP-CMR acquisitions to increase the in-plane spatial resolution (30–35), minimize dark-rim artifacts, improve the detection of subendocardial ischemia, and/or increase cardiac coverage and slice resolution [e.g., simultaneous multi-slice, SMS (36–40), or 3D whole-heart acquisitions (41–47)]. To minimize respiratory motion artifacts, motion compensation strategies (22, 48–51) and non-Cartesian sampling schemes (43, 44, 52–54) have been proposed. However, FB-FOSTERS offers several advantages. FOSTERS estimates rigid in-plane respiratory motion from the fat-only image, while most methods use the dynamic contrast-enhanced time series, which is prone to image registration errors due to changes in image intensity during contrast passage (13, 16, 22, 55, 56). Moreover, FOSTERS also eliminates signal contributions from the chest and body fat that have detrimental effects on motion estimation and MBF quantification (19). Incomplete fat suppression can lead to partial volume effects at the myocardial-epicardial border, which affects the MBF quantification accuracy. FOSTERS corrects for in-plane rigid motion during the inline reconstruction, thus avoiding geometric distortions and blurring caused by non-rigid methods in the presence of large respiratory displacements. The reconstructed FB-FOSTERS images show very small residual motion such that non-rigid motion registration can be successfully and efficiently applied before MBF quantification (12). A further benefit of FOSTERS is the inclusion of a low-resolution, low-saturation-time slice to measure the AIF for accurate MBF quantification (20). The low-saturation-time slice was used to account for T_1_ and T${}_{2}^{*}$-related signal loss in the AIF, in addition to the high-resolution dual-echo images. Furthermore, FOSTERS can be combined with non-Cartesian sampling, SMS, and 3D whole-heart acquisitions, which will be the focus of future work.

FOSTERS shows promise for future clinical stress/rest perfusion studies due to its robustness to in-plane motion, high IQ, as well as inline reconstruction implementation. The short acquisition window, together with the removal of breath-holding, can enable acquisition in patients with short RR intervals. In this study, only rest FFP-CMR scans were performed, but future studies will aim to validate FOSTERS in a larger cohort of patients with coronary artery disease during stress and rest FPP-CMR.

This study has several limitations that warrant discussion. First, 2D imaging was used and no through-plane motion correction was performed, which may influence the estimated MBF values. However, in this patient group, no severe through-plane motion was identified in FB-FOSTERS acquisitions, likely associated with the regularity of shallower breathing - reducing the risk of motion that can occur at the start or end of a long BH. In addition, in this patient group, sufficient fat content was present in the fat-only images, but more studies are warranted to assess the performance of the motion estimation in patients with low levels of fat around the heart. Additionally in future work, FOSTERS should be tested in a heterogeneous patient cohort that includes different patient profiles, to assess the impact of subcutaneous, epicardial, or intramyocardial fat content. Due to practical reasons, FOSTERS and standard BH scans were acquired with a relatively short pause between scans (5–7 min), so baseline contrast contamination was observed in some standard BH datasets. In addition, a randomized order of sequences was not performed, with FOSTERS always preceding the standard BH FPP-CMR images. Finally, myocardial coverage was limited to three slices, but whole-heart coverage could be of high diagnostic utility (46).

## Conclusion

FOSTERS, a k-t accelerated dual-saturation dual-echo Dixon FPP-CMR framework, enables free-breathing and high-resolution quantitative FPP-CMR and improved MBF quantification, with automatic in-plane respiratory motion correction and T${}_{2}^{*}$ correction. When compared to standard-resolution breath-hold FPP-CMR, no statistical differences were found in the image quality score, and substantially reduced dark-rim artifacts were observed in the FOSTERS FPP-CMR images. Future studies will aim to test FOSTERS in patients with coronary artery disease during stress.

## Data Availability Statement

The raw data supporting the conclusions of this article will be made available by the authors, without undue reservation.

## Ethics Statement

The studies involving human participants were reviewed and approved by National Research Ethics Service (15/NS/0030). The patients/participants provided their written informed consent to participate in this study.

## Author Contributions

JT and CMS implemented the sequence and the reconstruction framework, performed data analysis and interpretation, statistical analysis, and drafted the main manuscript. TS contributed to study design and contributed to pulse sequence and reconstruction algorithms. EA, RC, FB, and AC contributed to patient recruitment, CMR imaging and performed image quality scoring. JS-G, MD, CS, JM, JK, JS, and MB contributed to pulse sequence and reconstruction algorithms. MH and TC contributed substantially to the conception and design of the study, data analysis and interpretation, and drafted the main manuscript. All authors read, revised, and approved the final manuscript.

## Funding

This work was supported by the Wellcome/EPSRC Center for Medical Engineering [WT 203148/Z/16/Z], the Swedish Research Council [grant 2018-04164], and the European Commission within the Horizon 2020 Framework through the MSCA-ITN-ETN European Training Networks (project number 642458).

## Conflict of Interest

JT, TS, JS-G, JS, and MB are Philips Healthcare employees. MD, CS, JM, and JK are employees of Philips Research Europe. The remaining authors declare that the research was conducted in the absence of any commercial or financial relationships that could be construed as a potential conflict of interest.

## Publisher's Note

All claims expressed in this article are solely those of the authors and do not necessarily represent those of their affiliated organizations, or those of the publisher, the editors and the reviewers. Any product that may be evaluated in this article, or claim that may be made by its manufacturer, is not guaranteed or endorsed by the publisher.

## Supplementary Material

Supplementary Table 1 Patients Characteristics.

Supplementary Video 1 First-pass perfusion cardiac MR obtained from patient 5 using FOSTERS, CS, and standard BH.

Supplementary Video 2 First-pass perfusion cardiac MR obtained from patient 7 using FOSTERS, CS, and standard BH.

Supplementary Video 3 Fat- and water-only dynamic images were obtained from one representative patient using FOSTERS and CS. A temporal profile in the foot-head direction (yellow dashed line) is also shown.

Supplementary Figure 1 Example of myocardium-to-blood border sharpness analysis in the mid-short-axis slices of two representative patients. One profile (red line) is manually drawn perpendicular to the myocardium-blood interface, avoiding myocardial trabeculations. Image sharpness is defined as the pixel distance (d) between 20% and 80% of the maximum intensity range (r). For each approach (FOSTERS, CS, and standard BH), the final image sharpness value was calculated as the average sharpness across the three acquired slices.

Supplementary Figure 2 Fat- and water-only dynamic images were obtained from one representative patient using FOSTERS and spatial wavelet-based compressed sensing (CS). A single slice (mid-slice) for three different dynamic timeframes and a temporal profile in the foot-head direction (yellow dashed line) are shown. The FOSTERS fat-only images contain enough structural information to allow in-plane motion estimation and were less affected by the contrast bolus. The CS fat-only images still contain a high level of noise which hampers the motion estimation performance resulting in lower quality water-only images. Supplementary Video 3 contains an animation of this dataset for both approaches.
