# Supplementary figures and images for: High-Resolution Free-Breathing Quantitative First-Pass Perfusion Cardiac MR Using Dual-Echo Dixon With Spatio-Temporal Acceleration
Source: Front Cardiovasc Med. 2022 Apr 29;9:884221. doi: 10.3389/fcvm.2022.884221 (PMC9099052; doi:10.3389/fcvm.2022.884221)

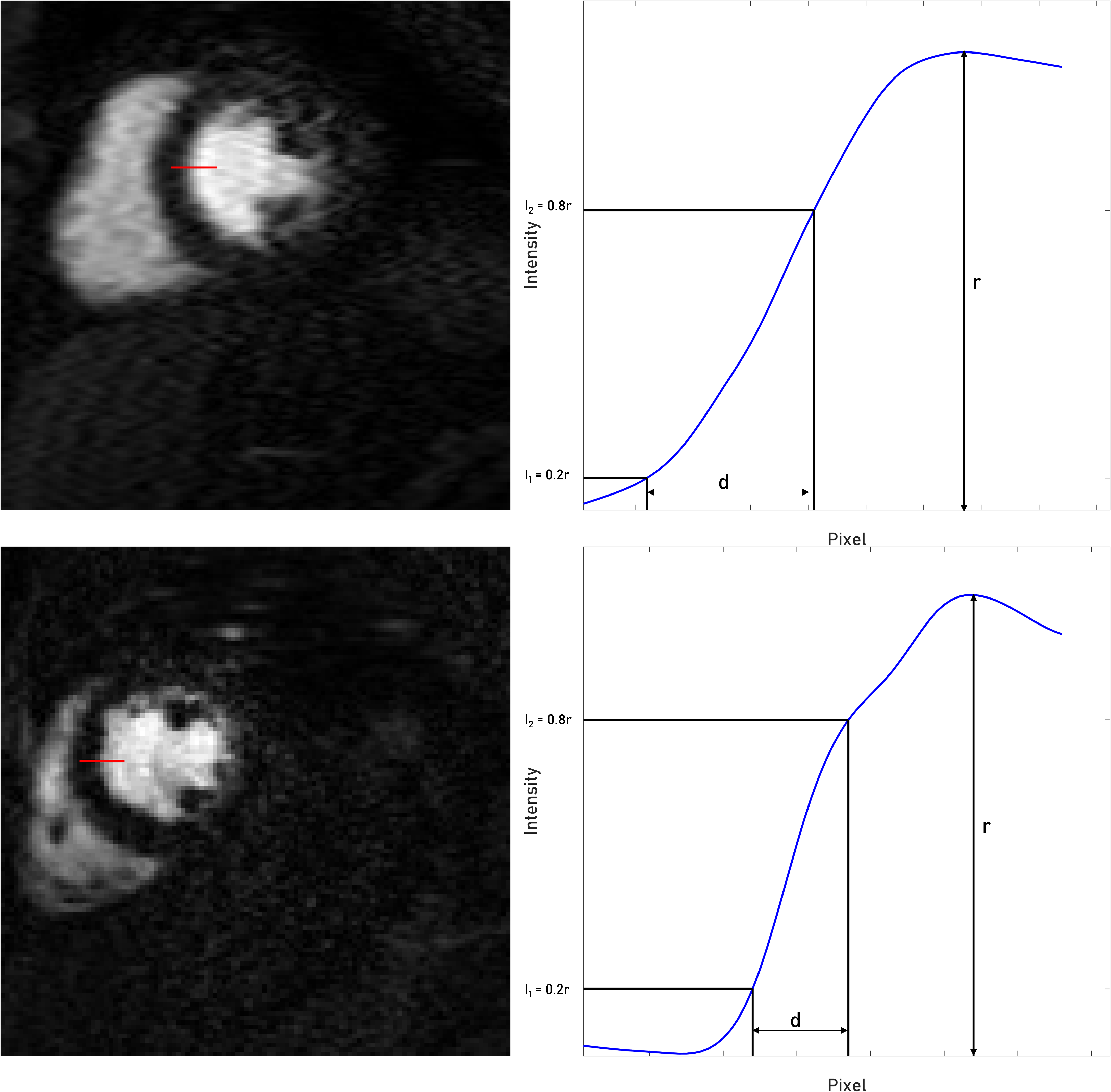

Supplement: Supplementary Figure 1 — Example of myocardium-to-blood border sharpness analysis in the mid-short-axis slices of two representative patients. One profile (red line) is manually drawn perpendicular to the myocardium-blood interface, avoiding myocardial trabeculations. Image sharpness is defined as the pixel distance (d) between 20% and 80% of the maximum intensity range (r). For each approach (FOSTERS, CS, and standard BH), the final image sharpness value was calculated as the average sharpness across the three acquired slices. [file Image_1.TIF]

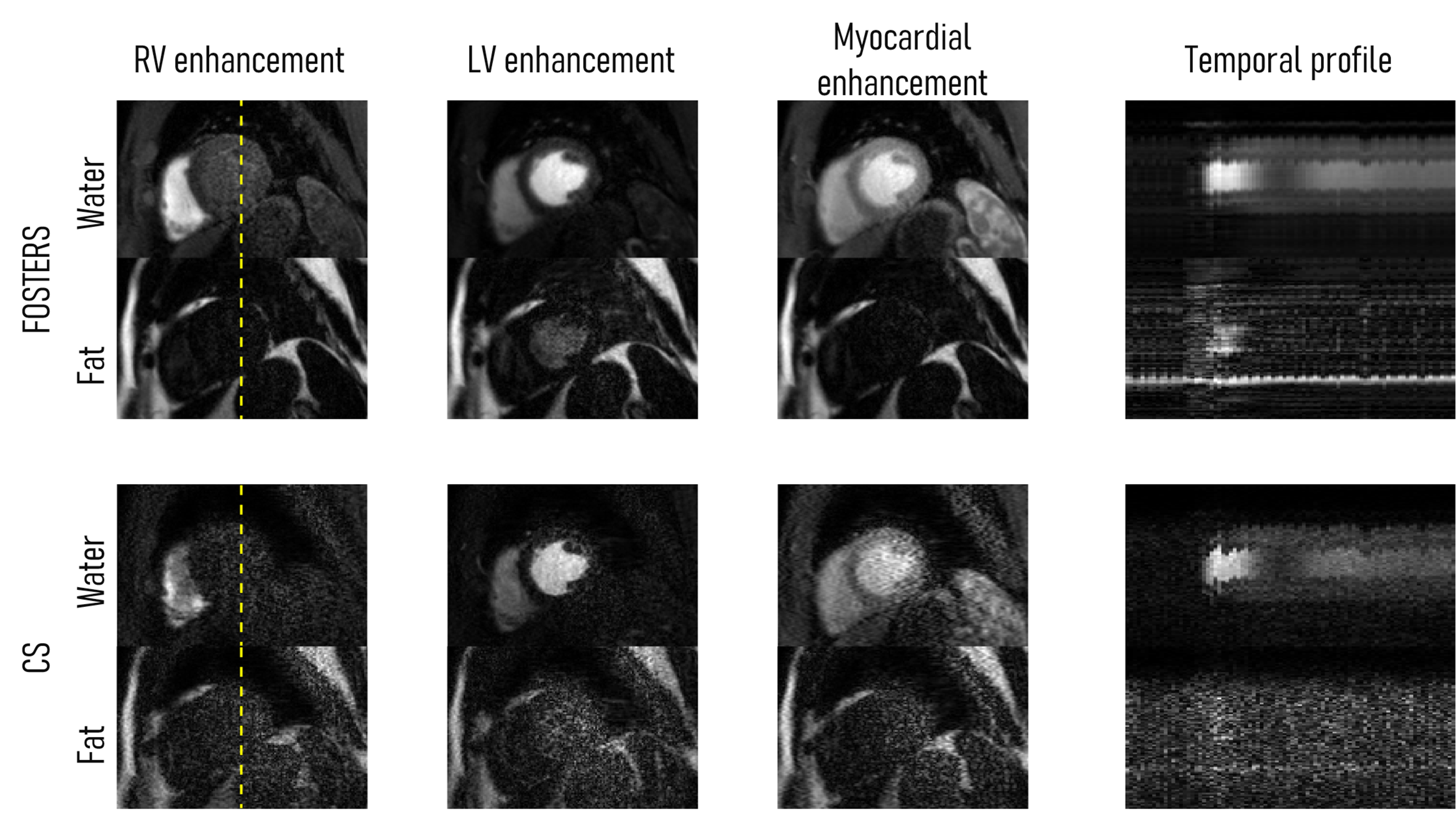

Supplement: Supplementary Figure 2 — Fat- and water-only dynamic images were obtained from one representative patient using FOSTERS and spatial wavelet-based compressed sensing (CS). A single slice (mid-slice) for three different dynamic timeframes and a temporal profile in the foot-head direction (yellow dashed line) are shown. The FOSTERS fat-only images contain enough structural information to allow in-plane motion estimation and were less affected by the contrast bolus. The CS fat-only images still contain a high level of noise which hampers the motion estimation performance resulting in lower quality water-only images. Supplementary Video 3 contains an animation of this dataset for both approaches. [file Image_2.PNG]
